# Identification of E3 Ubiquitin Ligases Associated with Survival in Soft Tissue Sarcomas

**DOI:** 10.3390/ijms27146350

**Published:** 2026-07-17

**Authors:** Zackary Bender, Hannah C. Beird, Peter Larsen, Gary S. Coombs

**Affiliations:** 1 Biology Department, Waldorf University, 106 South 6th Street, Forest City, IA 50436, USA; zackbender03@gmail.com; 2 Department of Genomic Medicine, The University of Texas, UT MD Anderson Cancer Center, 1881 East Road, Houston, TX 77054, USA; hccheung@mdanderson.org; 3 MercyOne North Iowa Medical Center, 1000 4th Street SW, Mason City, IA 50401, USA; peterlarsen@mercyhealth.com

**Keywords:** E3 ubiquitin ligase, BCR regulation, telomere end packaging, SIRT1, chromosome condensation, HUSH complex

## Abstract

Proteasome inhibitors are approved to treat multiple myeloma and mantle cell lymphoma. Recent reports suggest sarcomas also display proteasome addiction. Mechanistic explanations cite proteotoxic stress. In sarcoma patients, we analyzed the impacts of 377 human E3 ubiquitin ligases on sarcoma patient overall survival (OS) and recurrence-free survival (RFS), identified substrates of E3 ligases with the most significant and robust effects, and performed enrichment analyses. High expression of 102 E3 ligases was associated with shortened OS. Thirteen of these shortened OS by >40 months, six with false-discovery rates (FDR) ≤ 5%. Nineteen showed correlation between increased expression and shortened RFS, two with FDR ≤ 5%. Overexpression of 73 E3 ligases significantly extended OS, with 18 extending OS by >40 months; six with FDR ≤ 5%. Elevated expression of 21 significantly extended RFS, one with FDR ≤ 5%. Enrichment analyses of substrates unique to the E3 ligases whose elevated expression most reliably shortened or extended OS by >40 months revealed non-overlapping functions: the E3 ligases associated with shortened OS uniquely targeted cell cycle, cell–cell communication, cellular responses to stimuli, chromatin organization, DNA repair, DNA replication, hemostasis, reproduction, and vesicle-mediated transport functions. Both OS-impacting E3 ligase sets targeted developmental biology, gene expression, immune system, metabolism of proteins, and signal transduction functions. Three specific functions were targeted by both groups. Functions uniquely targeted by each set of ligases could reveal therapeutic targets with a greater therapeutic index than the proteasome.

## 1. Introduction

Sarcomas are a broad group of over 70 types of neoplasms originating in mesenchymal tissues derived from embryonic mesoderm layer cells [1,2]. Approximately 80% of sarcomas originate in soft tissues including connective tissues, blood vessels, fat, and smooth and skeletal muscle. The remaining 20% arise in bone or cartilage [3]. Age of onset varies with sarcoma type, but cases are most prevalent in children, adolescents, and young adults under 30 years of age [4,5]. Roughly 15% of all cancers in children are sarcomas [6]. Roughly 17,000 new cases of sarcoma were diagnosed in the United States in 2023, with approximately 13,460 of these being soft tissue sarcomas [7]. Approximately 7280 deaths occurred in the United States in 2023 due to all types of sarcomas [8]. First-line treatment for sarcomas is typically surgery with or without chemotherapy and/or radiation therapy [9]. In gastrointestinal stromal tumors, imatinib (which inhibits c-KIT) is the first line treatment option [9]. For locally advanced or metastatic sarcomas, targeted therapeutics or immunotherapies may be administered as first line treatment on a case-by-case basis [10,11]. Five-year survival rates for soft tissue sarcoma are 81% for localized, 56% for regional, and 16% for distant metastatic disease. Definitive diagnosis is often slow due to the rarity of these cancers, subtle and slowly progressive symptoms, and the similarity of these symptoms to those of less serious conditions [12]. Delays in diagnosis result in low rates of treatment at the localized stage, reflected in the 65% 5-year survival rate for sarcomas overall, making sarcomas among the deadliest of rare cancers and illustrating the need for effective new therapy options.

**Figure 1:**
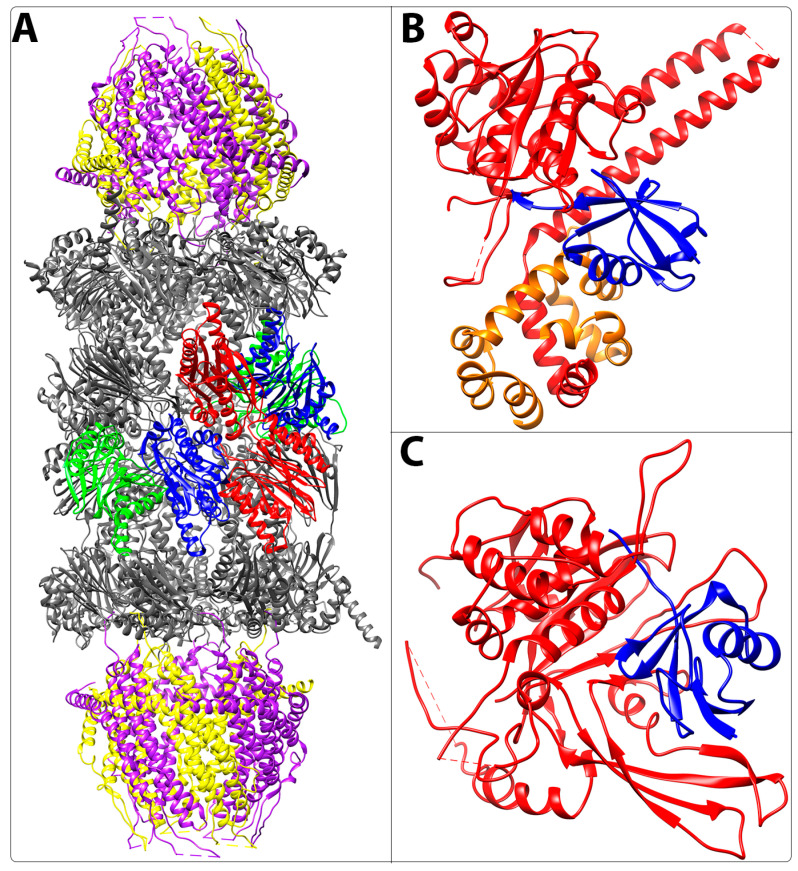
(**A**) structure of the core proteasome capped by PA28 (11S) proteasome activator complexes (PDB ID 7NAP). This complex, known as the immunoproteasome, lacks ATPase activity and plays an essential role in producing antigenic peptides for MHC class I molecule display. The a and b subunits of the 11S proteasome activator complex (respectively encoded by PSME1 and PSME2 genes) are displayed in yellow and purple, respectively. In the core proteasome, the β1 β2 and β5 subunits possess threonine protease active sites, with β1 (green) cutting peptide bonds C terminal to acidic amino acids, β2 (red) cutting C terminal to basic amino acids, and β5 (blue) cutting C terminal to large hydrophobic amino acids. Inhibitors of proteasome proteolytic activity include peptide boronic acids (bortezomib, ixazomib, delanzomib), peptide epoxyketones (carfilzomib, epoxomicin), peptide aldehydes (MG132, MG115), and the natural product lactacystin. (**B**) Structure of ubiquitin carboxyl terminal hydrolase isoform L5 (red), encoded by uchl5, in complex with ubiquitin (blue) and the RPN13 DEUBAD domain (gold) (PDB ID 4UEL). (**C**) Structure of ubiquitin specific peptidase 14 (red), encoded by usp14, in complex with ubiquitin (blue) (PDB ID 2AYO). The ubiquitin hydrolases in (**B**,**C**) are essential for protein degradation by the proteasome and can be inhibited with small molecule inhibitors including b-AP15, VLX1570, and auranofin. Molecular graphics and analyses performed with UCSF Chimera, developed by the Resource for Biocomputing, Visualization, and Informatics at the University of California, San Francisco, with support from NIH P41-GM103311.

The proteasome inhibitor bortezomib received FDA approval for treatment of multiple myeloma in 2003 and for treatment of mantle cell lymphoma in 2006. Investigations, including clinical trials, are underway to characterize the efficacy of proteasome inhibition in non-small cell lung carcinoma; breast, prostate, and ovarian cancer; and various other solid tumor types. A recent Ewing sarcoma cell-based screen for small molecules that could inhibit proliferation revealed a class of benzyl-4-piperidone compounds that can selectively inhibit proliferation of multiple Ewing sarcoma cell lines [13]. These compounds were shown to inhibit 19S proteasome cap function by inhibiting its integral deubiquitinating enzymes USP14 and UCHL5 [13]. Specific proteasome dependence in Ewing sarcoma was also indicated by a genome-wide gene knockdown screen using shRNA [13]. In addition, treatment of osteosarcoma cells with bortezomib inhibited cell proliferation and induced apoptosis via a mechanism involving upregulation of the osteoblastic differentiation factor Runx2 [14]. Further, proteasome activator complex subunit 2 (PSME2) was found to inhibit osteosarcoma cell proliferation, migration, and invasion, suggesting another mechanism of proteasome addiction [15]. Some evidence also exists for proteasome addiction in soft tissue sarcomas. The proteasome inhibitor MG132 dose dependently increased apoptosis and antagonized proliferation in three uterine leiomyosarcoma cell lines and induced G2/M phase arrest in two of these cell lines [16], and high nuclear expression of proteasome activator complex subunit 1 (PSME1) was associated with poor survival in soft tissue leiomyosarcoma patients [17]. An overview of proteasome structure, inhibitable enzymatic functions, and proteasome interaction with the proteasome activator complex is provided in Figure 1 [18,19,20].

To better understand mechanism(s) of proteasome addiction in soft tissue sarcomas, we investigated the impact of each of 377 ubiquitin ligases, in a list curated by the Epithelial Systems Biology Laboratory of the National Heart Lung and Blood Institute [21] on overall survival (OS) among a cohort of 259 soft tissue sarcoma patients using data from The Cancer Genome Atlas (TCGA). This cohort includes patients with leiomyosarcoma, dedifferentiated liposarcoma, undifferentiated pleomorphic sarcoma, myxofibrosarcoma, synovial sarcoma, and malignant peripheral nerve sheath tumors [22]. For E3 ligases whose expression level was significantly associated with an OS difference of >40 months and had false-discovery rates (FDR) ≤ 5%, we identified sets of known and predicted substrates using Ubibrowser 2.0 [23] and performed enrichment analyses to learn what cellular functions are potentially modulated by proteasomal activity guided by these relevant E3 ligases.

## 2. Results

### 2.1. Impacts of E3 Ubiquitin Ligases on Overall Survival

**Figure 2:**
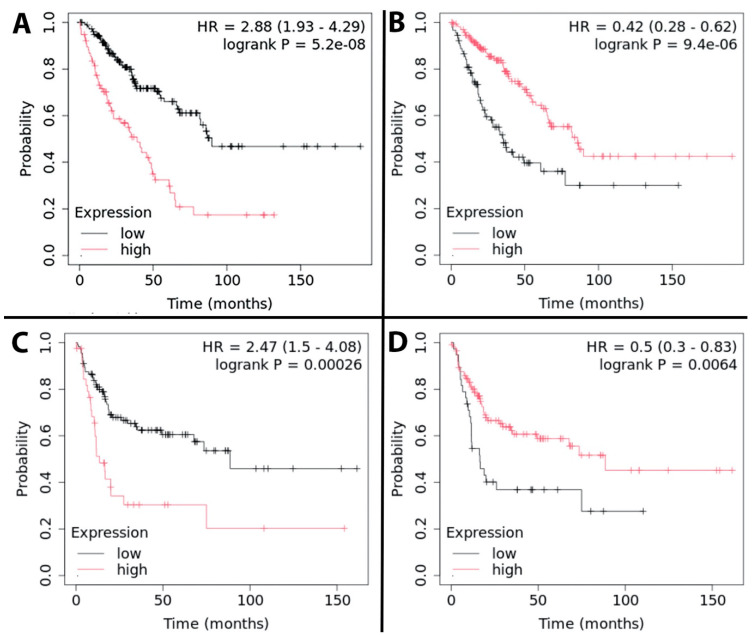
Kaplan–Meier plots of the E3 ubiquitin ligase expression groups with the largest impacts on survival. (**A**) High expression of TTC3 was associated with shortened median overall survival (OS) by 50.63 months. (**B**) High expression of VPS18 was associated with extended median OS by 50.86 months. (**C**) High expression of MNAT1 was associated with shortened median recurrence-free survival (RFS) by 75.03 months. (**D**) High expression of TRIM10 was associated with extended median RFS by 72.10 months.

We identified very small subsets of ubiquitin ligases whose high expression was significantly associated with extended or shortened overall survival (OS). TTC3 exerted the strongest negative impact on OS, and VPS18 exerted the strongest positive impact on OS (Figure 2A,B).

**Figure 3:**
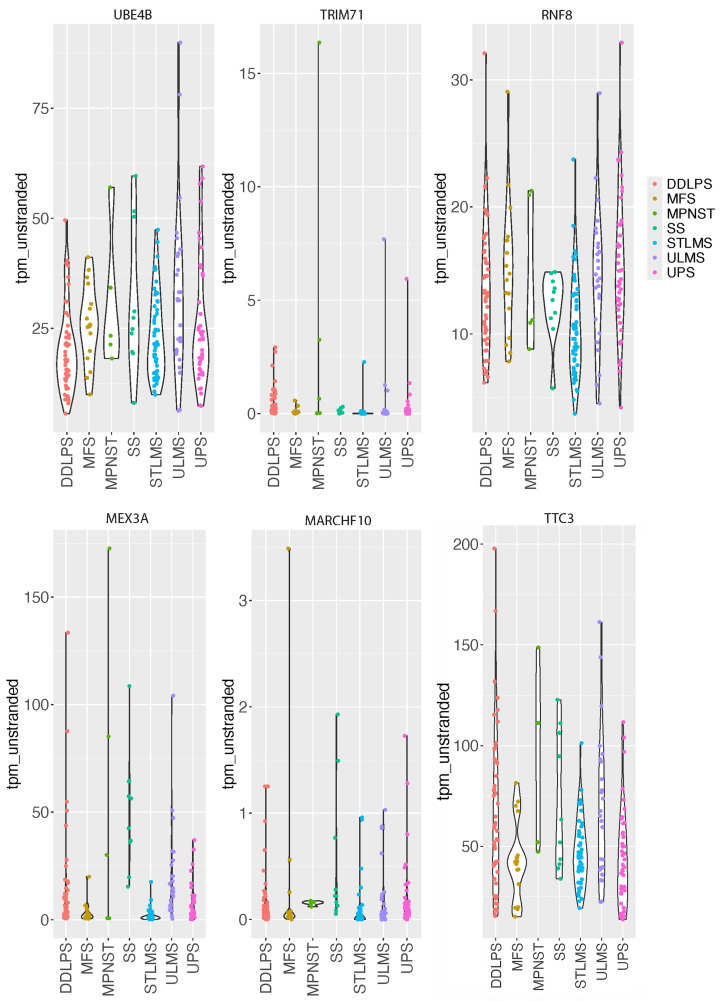
Violin plots of transcripts per million (TPM) values (Y-axis) for E3 ubiquitin ligases whose up regulation was associated with shortened overall survival among sarcoma patients by >40 months and FDR ≤ 5% according to sarcoma histologic type (Table 1). DDLPS = dedifferentiated liposarcoma; MFS = myxofibrosarcoma; MPNST = malignant peripheral neural sheath tumor; SS = synovial sarcoma; STLMS = soft tissue leiomyosarcoma; ULMS = uterine leiomyosarcoma; UPS = undifferentiated pleomorphic sarcoma.

Among the 377 human E3 ubiquitin ligases analyzed, 102 (27%) ligases showed a significant association between high expression and shortened OS, with *p*-values ranging from 0.05 to 5.2 × 10^−8^, and impacts on survival ranging from 6.2 to 50.6 months (median 31.6 ± 9.2). Thirteen of these ligases (see Table 1) were associated with OS decreases of >40 months. Eight of these thirteen remained significantly associated with OS when median transcripts per million (TPM) was used as the cutoff. Six of these had an FDR ≤ 5%. Violin plots for each of the 13 E3 ligases associated with >40 months shorter OS indicate that each sarcoma histologic type was usually represented in both high and low expression groups (Figure 3 and Figure S1). However, CCNB1IP1 expression was relatively high in all synovial sarcoma patients, and PCGF3 expression was relatively low in all myxofibrosarcoma patients. Violin plots also reveal very low expression of TRIM71 and MARCHF10 in all patients, with median TPM below 2 for each ligase.

**Figure 4:**
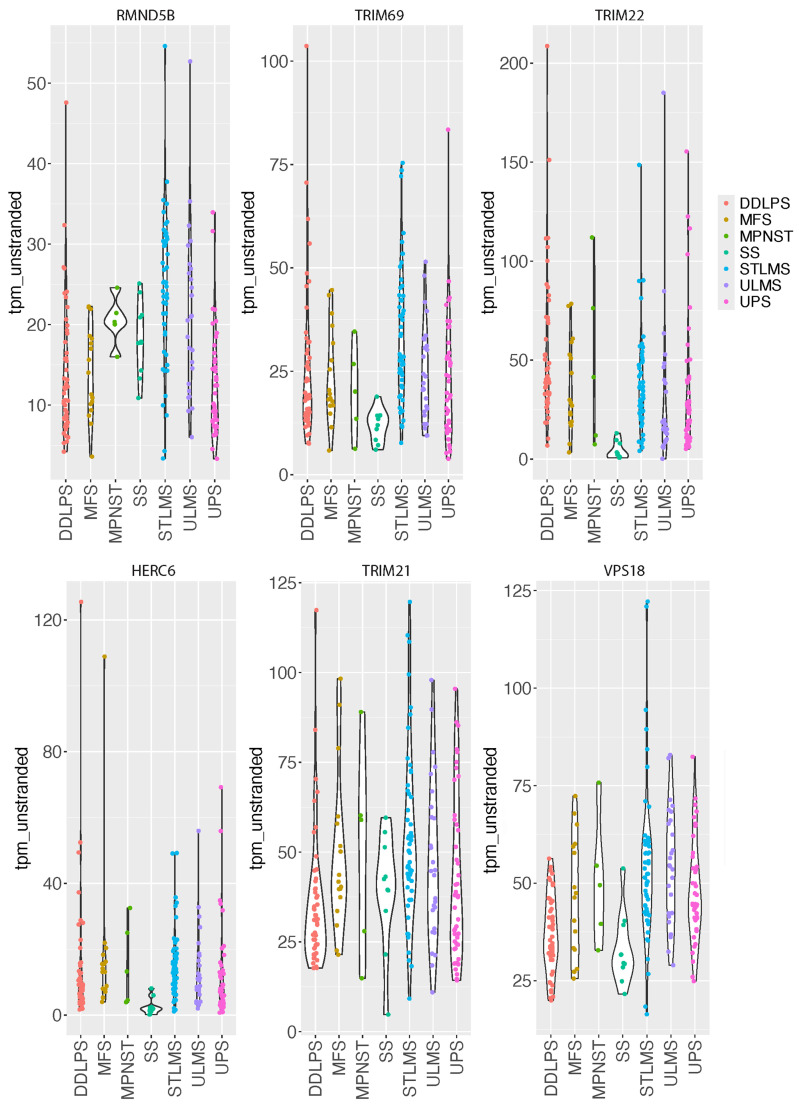
Violin plots of TPM values (Y-axis) for E3 ubiquitin ligases whose upregulation was associated with extended overall survival among sarcoma patients by >40 months and FDR ≤ 5% according to sarcoma histologic type (Table 2). DDLPS = dedifferentiated liposarcoma; MFS = myxofibrosarcoma; MPNST = malignant peripheral neural sheath tumor; SS = synovial sarcoma; STLMS = soft tissue leiomyosarcoma; ULMS = uterine leiomyosarcoma; UPS = undifferentiated pleomorphic sarcoma.

A separate set of 73 E3 ligases (19.4%) showed a significant association between high expression and extended OS, with *p*-values ranging from 0.049 to 9.4 × 10^−6^, and impacts on survival ranging from 3.1 to 50.8 months (median 32.4 ± 9.1). Eighteen of these ligases (Table 2) were associated with OS increases of >40 months. Only two of these ligases (RMND5B and TRIM21) remained significantly associated with OS when median TPM was used as the cutoff, but six of them revealed an FDR ≤ 5% when an optimal cutoff was selected using COX analysis. For the 18 E3 ligases associated with OS increases of >40 months, each histologic type was usually represented in both high and low expression groups (Figure 4 and Figure S2). However, eight of these eighteen ligases displayed uniformly low expression in synovial sarcoma, and SYVN1 displayed uniformly high expression in malignant peripheral nerve sheath tumors (MPNST).

### 2.2. Impacts of E3 Ubiquitin Ligases on Patient Recurrence-Free Survival

In total, 102 E3 ligases displayed a positive association between elevated expression and shortened OS, and 73 E3 ligases displayed a positive association between elevated expression and extended OS. We next assessed each of these E3 ligases for the impact of its expression level on recurrence-free survival (RFS). MNAT1 exerted the largest negative impact on RFS, and TRIM10 exerted the largest positive impact on RFS (Figure 2C,D).

Among the E3 ligases whose elevated expression was associated with shortened OS, 15 (15%) were also significantly associated with shortened RFS (see Table 3). Among these, nine showed median RFS decreases of 56–75 months, and six showed median RFS decreases of 3–10 months. Three of the nine E3 ligases with large survival impacts (RNF26, TRAF4, and UHRF1) remained significant when median TPM was used as a cutoff. Two E3 ligases (MNAT1 and UHRF1) yielded FDR ≤ 5% when an optimal cutoff was selected using COX analysis. Violin plots showed that each histologic type was usually represented in both high and low expression groups (Figure S3). However, RAD18 was uniformly low in synovial sarcoma, and MEX3C and MKRN2 were uniformly high in MPNST.

Among the E3 ligases whose elevated expression was associated with extended OS, 12 (16%) were also significantly associated with extended RFS (Table 4). Among these, 11 showed median RFS increases of 48.8–72.1 months (only TRIM8 showed a small difference of 1.9 months). Only one of the eleven E3 ligases with large survival impacts (RMND5B) was validated as still significant when using median TPM as a cutoff. Similarly, only one E3 ligase (TMEM129) yielded an FDR ≤ 5% when an optimal cutoff was selected using COX analysis. Violin plots again indicate that each histologic type was usually represented in both high and low expression groups (Figure S4). However, RNF130 expression skewed high in MPNST, and TRIM8 expression skewed low in synovial sarcoma.

### 2.3. Putative Substrate Pools of E3 Ligases That Significantly Impact Overall Survival

For each of the six E3 ligases whose elevated expression was significantly associated with shortened OS with a decrease of >40 months and an FDR ≤ 5%, known and predicted substrates were identified using UbiBrowser 2.0. Duplicate proteins and proteins shared with the substrates of the E3 ligases whose expression is associated with extended OS were eliminated, resulting in a pool of 844 plausible unique substrates (36 known and 808 predicted). UbiBrowser 2.0 was also used to identify known and predicted substrates for each of the six E3 ligases whose elevated expression was significantly associated with extended OS with an increase of >40 months and an FDR ≤ 5%. Duplicate proteins were eliminated, resulting in a pool of 182 plausible substrates (23 known and 159 predicted).

### 2.4. Enriched Functions Associated with E3 Ligases Whose Elevated Expression Is Associated with Shortened OS

**Figure 5:**
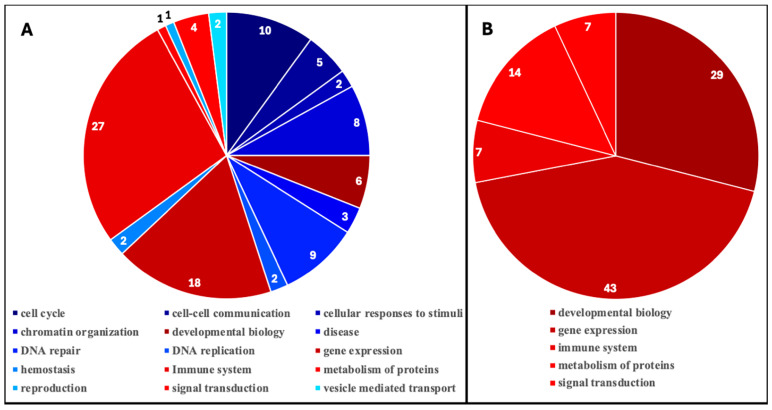
Highest hierarchical level categories of enriched functions found in Reactome associated with E3 ubiquitin ligases that impact overall survival of sarcoma patients. (**A**) Enriched function categories associated with substrates of E3 ligases whose overexpression was associated with significantly shortened sarcoma patient OS; categories unique to substrates of E3 ligases associated with diminished OS are represented in shades of blue. (**B**) enriched function categories associated with substrates of E3 ligases whose overexpression significantly extends sarcoma patient OS; Categories shared by both sets of substrates are represented in shades of red.

Elevated expression of the E3 ubiquitin ligases UBE4B, TRIM71, RNF8, MEX3A, MARCHF10, and TTC3 (see Table 1) was associated with significantly (*p* = 8.7 × 10^−4^ to 5.2 × 10^−8^; FDR = 2–1%, Hazard Ratio (HR) = 1.96–2.78) shortened OS by 40.1 to 50.6 months with FDR ≤ 5%, suggesting that these six E3 ligases make a proportionately large contribution to proteasome addiction in soft tissue sarcomas. Among the identified substrates of these ligases, 844 substrates were not also substrates of the E3 ligases associated with extended OS. Among the functions associated with these 844 substrates, 117 enriched functions (excluding those related to infectious disease) had *p* ≤ 1 × 10^−8^. Among these enriched functions, the most highly represented pathway category at the top of the Reactome hierarchy was immune system (32 enriched functions), followed by gene expression (21 enriched functions), cell cycle (12 enriched functions), chromatin organization and DNA repair (10 enriched functions each), developmental biology (7 enriched functions), cell–cell communication (6 enriched functions), signal transduction (5 enriched functions), and disease (4 enriched functions), as well as hemostasis, vesicle mediated transport, DNA replication, and cellular responses to stimuli (2 enriched functions each), and metabolism of proteins and reproduction (1 enriched function each) (Figure 5A). The top 25 results of enrichment analysis using Reactome (from all hierarchical levels), with the lowest *p*-values, are presented in Table 5.

### 2.5. Enriched Gene Sets Associated with E3 Ligases Whose Elevated Expression Is Associated with Extended OS

Elevated expression of the E3 ubiquitin ligases RMND5B, TRIM69, TRIM22, HERC6, TRIM21, and VPS18 (Table 2) was associated with significantly (*p* = 3.6 × 10^−4^ to 9.4 × 10^−6^; FDR = 5–1%, HR = 0.49–0.43) extended OS by >40 months with FDR ≤ 5%, suggesting that these E3 ligases target substrates that support tumor progression. Among the identified substrates of these ligases, 184 substrates were not also substrates of the E3 ligases associated with shortened OS. These 184 substrates had 14 enriched functions with *p* ≤ 1 × 10^−8^ (Table 6). The most highly represented pathway category at the top of the Reactome hierarchy was gene expression (6 uniquely enriched functions), followed by developmental biology (4 enriched functions), metabolism of proteins (2 functions), and immune system and signal transduction (1 function each) (Figure 5B).

## 3. Discussion

### 3.1. Substrates of E3 Ligases Whose Upregulation Shortened OS Are Enriched in Tumor Suppressor Functions

Among the 117 most significantly enriched functions associated with substrates of the E3 ligases whose elevated expression shortened OS, suggesting that they play key roles in proteasome addiction, 66 fall under overarching categories (immune system, chromatin organization, cell cycle, DNA repair and DNA replication) known to harbor tumor suppressors.

Twenty-one functions fall under gene expression, including six functions related to ribosomal RNA transcription by RNA polymerase I. Functional ribosomes are crucial for rapid growth and cell proliferation in sarcomas. RNA polymerase I is considered a potential therapeutic target in some sarcomas, with the RNA polymerase 1 inhibitor CX-5461 inducing an antiproliferative effect in SK-UT-1 leiomyosarcoma cells via G2 cell cycle arrest [24]. Six of the functions relate to epigenetic regulation of gene expression by mechanisms including DNA and histone methylation, histone acetylation, and chromatin modification. Loss-of-function alterations in epigenetic modulators are common in mesenchymal cancers and have been found in sarcomas including synovial, epithelioid, and Ewing sarcoma [25]. Another three gene expression functions involve RUNX1, which can act as an oncogene or a tumor suppressor depending on context. In the blood, it plays a role in proper differentiation of stem cells, and its loss or participation in a fusion protein is seen in various blood and gastrointestinal cancers [26,27,28].

Seven functions fall under developmental biology. These functions include nucleosome establishment in male pronuclei, maternal-to-zygotic transition, HOX gene activation, and axon guidance in nervous system development. Proteins involved in maternal-to-zygotic transition include epigenetic regulators involved in establishing totipotency in zygotes that can later function as tumor suppressors [29,30]. HOX genes can act as tumor suppressors in context-specific cancers. For instance, HOXA5 has been shown to function as a tumor suppressor in osteosarcoma [31]. Tumor innervation underlies aggressiveness, perineural invasion/metastasis, poor prognosis, and cancer-associated pain [32].

Eight functions fall under cell–cell communication. These include regulation of *CDH1* gene transcription, cell–cell junction organization, telomere maintenance, and histone arginine methylation. *CDH1* encodes E-cadherin, which is generally considered to be a tumor suppressor. E-cadherin is an important contributor to cell–cell junctions, and its loss allows tumor cells to detach from the primary tumor without undergoing anoikis, thereby facilitating metastasis [33]. Telomere maintenance via reactivation of telomerase expression is a hallmark cancer cell behavior in ≥80% of solid tumor types [34], conferring immortality upon cancer cells, allowing them to proliferate indefinitely without becoming senescent. Finally, dysregulation of protein arginine methyltransferases is linked to tumor initiation, progression, and therapeutic resistance [35].

**Figure 6:**
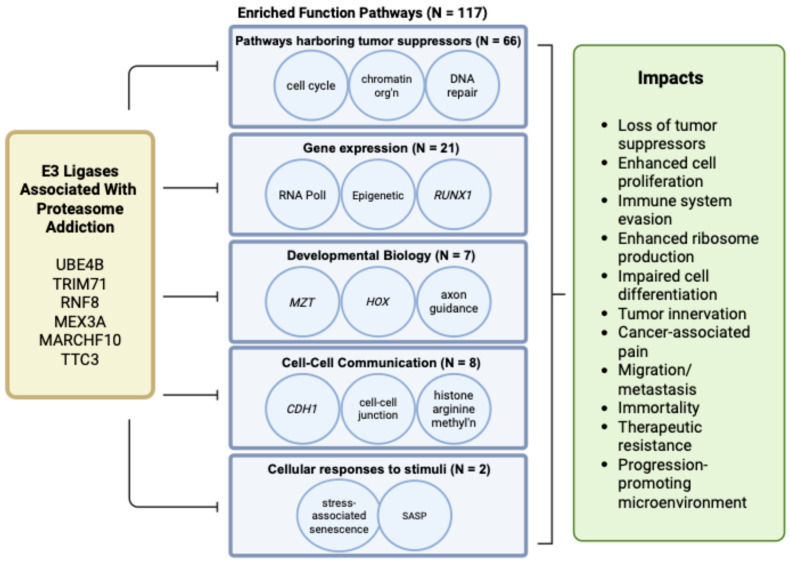
Scheme illustrating impacts on disease of pathways enriched in known and predicted substrates of the 6 E3 ligases predicted to strongly mediate proteasome addiction in soft tissue sarcomas. In total, 117 pathways are enriched with *p*-values ≤ 1 × 10^−8^. Among these, 66 enriched functions fall under categories (immune system, chromatin organization, cell cycle, DNA repair and DNA replication) known to harbor tumor suppressors, and 21 functions fall under “gene expression”, including functions essential for ribosome production, functions involved in epigenetic regulation of gene expression, and gene expression mediation by RUNX1. Seven functions fall under “developmental biology”, including maternal to zygotic transition, HOX gene regulation, and axon guidance. Eight functions are related to cell–cell communication. These include E-cadherin expression from the CDH1 gene, cell–cell junction formation, and histone arginine methylation. Two functions fall under cellular responses to stimuli. These are DNA damage/telomere stress-associated senescence and senescence-associated secretory phenotype (SASP). The inhibitory impact of the six listed E3 ligases on these enriched functions enhances tumor progression and aggressiveness and negatively impacts patient prognosis as described in the right-hand panel.

Two functions fall under cellular responses to stimuli: DNA damage/telomere stress-associated senescence and senescence-associated secretory phenotype. Senescence in cancer cells can halt tumor progression [36], although the senescence associated secretory phenotype can create a microenvironment that fosters cancer growth and progression [37]. A schematic of enriched functions among substrates of E3 ligases associated with shortened OS and their impacts on cancer is presented in Figure 6.

### 3.2. Substrates of E3 Ligases Whose Upregulation Extended OS Are Enriched in Oncogenic Functions

The 184 known and predicted substrates of the six E3 ligases whose upregulation extended OS included 14 enriched functions with *p* ≤ 1 × 10^−8^ (Table 6). These functions fall under the overarching categories of gene expression, developmental biology, metabolism of proteins, immune system, and signal transduction. Only three of these fourteen functions were also enriched among the substrate pool associated with shortened OS: cytokine signaling in immune system, activation of HOX genes during differentiation, and activation of anterior HOX genes in hindbrain development during early embryogenesis.

The most highly represented category of unique functions was gene expression. These functions included RNA polymerase II-mediated transcription, TP53-regulated transcription, and FOXO-regulated transcription. While cancer cell dependence on RNA polymerase II is well established, it is curious that TP53 and FOXO family transcription factor functions were enriched among substrates of E3 ligases associated with extended OS, as both TP53 and FOXO are established tumor suppressors [38].

It is also curious that the primary enriched function under developmental biology was differentiation of naïve CD4^+^ T helper 2 cells (Th2 cells), as these cells are traditionally associated with tumor progression and immune function evasion. However, recent research has revealed their potential to directly orchestrate tumor necrosis through recruitment of pro-inflammatory cells [39,40].

The enriched functions under the category of metabolism of proteins both relate to E3 ligase-mediated protein SUMOylation. This could potentially explain the enrichment of tumor-suppressive functions among the substrates of OS-extending E3 ligases. For instance, FOXO SUMOylation can serve as a switch, impacting FOXO stability, subcellular localization, and transcriptional activity. Its hyper-SUMOylation in rhabdomyosarcoma promotes tumor cell survival and proliferation [41]. In addition, TP53 SUMOylation can act as a switch promoting relocalization of nuclear TP53 to the cytoplasm [42].

The one enriched function under signal transduction was “PIP3 activates AKT signaling”. AKT signaling is a well-established promoter of tumor growth, aggressiveness, and therapeutic resistance in many cancers, including soft tissue sarcomas [43,44].

### 3.3. Adverse Events Associated with Proteasome Inhibitor Therapy

Real-world data associate proteasome inhibitor therapy with cardiovascular adverse events including heart failure, hypertension, arrhythmias, and ischemic heart disease including acute coronary syndrome and angina. Proteasome inhibitors can also negatively impact blood cell counts and cause gastrointestinal symptoms, including nausea, vomiting, diarrhea, and constipation [45]. Rates of serious adverse events associated with proteasome inhibitors can be substantial, though they vary among agents; for instance, cardiovascular adverse events associated with carfilzomib, bortezomib, and ixazomib occurred with respective frequencies of 7–27%, 0.6–4.1%, and 1.3% [46].

Other possible unintended effects of proteasome inhibitors involve the essential role of proteasomes in circadian clock function. Proteasomes degrade core clock proteins, including period and cryptochrome proteins, at specific times within the circadian period, allowing the clock to reset. Thus, proteasomes are essential for persistence of the circadian clock over multiple cycles and impact the length of a circadian period. Further, growing evidence indicates that a functional circadian clock exerts potent tumor suppressive activity, impacting immune functions, metabolism, cell cycle control, and DNA repair [47]. It is thus possible that proteasome inhibitor therapy could exert oncogenic effects that counterbalance its therapeutic effects in cancer patients.

The known and potential adverse events associated with proteasome inhibition represent a need for novel targets with similar efficacy but fewer or milder adverse effects, which could be identified through a better understanding of proteasome addiction through upstream analysis of key ubiquitin ligases and their target proteins and processes. Proteotoxic stress is frequently cited as the primary mechanism of proteasome addiction in cancers [48,49]. Proteotoxic stress is characterized by protein misfolding and aggregation, which can be elevated in cancers involving a high percentage of the proteome for several reasons, including high expression and translation rates (which both increase error rates in translation) and accumulation of mutations that impact folding kinetics, stability of folded proteins, and ability to form physiologically important complexes [50]. Our results indicate that only 1.6% (6/377) of the human repertoire of E3 ubiquitin ligases, and 10.5% (844/8047) of known and predicted substrates of the 31 E3 ubiquitin ligases that significantly increased or decreased OS by >40 months impactfully contribute to proteasome addiction mechanisms. These results suggest that mechanisms with greater specificity than proteotoxic stress may contribute to proteasome addiction.

### 3.4. Anomalous E3 Ligase Expression Patterns

Among the E3 ligases that shortened OS and had an FDR of ≤5% and/or remained significant with median expression as the cutoff, PCGF3, MSL2, MARCHF5, UBE4B, and TTC3 expression skewed high in the MPNST histologic type. It is possible that this finding simply reflects a low sample number, as only five MPNST patients were included. If this skewed expression is broadly representative, these E3 ligases may not be useful for prognosis specifically in MPNST but could be a useful prognostic signature in the other six soft tissue sarcomas. Given that expression skewed high in MPNST for these E3 ligases, they may still have therapeutic value. Among the E3 ligases that extended OS and had FDR of ≤5% and/or remained significant with median as cutoff, TRIM22, TRIM69, and HERC6 all skewed low in expression in synovial sarcoma, suggesting they may lack prognostic potential specifically in synovial sarcoma. Among the E3 ligases that shortened RFS and had an FDR of ≤5% and/or remained significant with median as cutoff, UHRF1 expression skewed low and MNAT1 skewed high in synovial sarcoma, suggesting that both may lack prognostic value in synovial sarcoma, but MNAT1 may represent a valid therapeutic target. Among the E3 ligases that extended RFS and had FDR of ≤5% and/or remained significant with median as cutoff, TMEM129 expression skewed high in synovial sarcoma, suggesting that it may lack prognostic potential specifically in synovial sarcoma.

### 3.5. Novelty of These Systematic Analyses of E3 Ubiquitin Ligase Impacts on Survival in Soft Tissue Sarcoma Patients

To our knowledge, while several studies of individual E3 ubiquitin ligases impacts in specific soft tissue sarcomas exist in the literature, this is the first effort to systematically characterize the impact of each human E3 ubiquitin ligase on survival among soft tissue sarcoma patients. E3 ubiquitin ligases can also mediate SUMOylation, and Codenotti et al. [51] showed that the small-molecule SUMOylation inhibitor TAK-981 suppressed rhabdomyosarcoma cell proliferation and migration and enhanced the cytotoxic effects of co-administered chemotherapeutics. Another small body of research has investigated the oncogenic impacts of the E3 ligase UBR5 on tumor microenvironment and immune response in MPNST [52]. Shu et al. [53] used the same TCGA dataset used in our studies to specifically assess the prognostic value of UHRF1 in soft tissue sarcomas. They found a significant negative impact of elevated UHRF1 expression on OS and correlations with tumor necrosis, histological type, and metastasis. Extending beyond soft tissue sarcomas, TRIM8 expression has been shown to modulate EWS/FLI fusion levels, exerting a positive effect on survival in Ewing sarcoma [54], and TRIM22 leads to NRF2 degradation, thereby promoting ROS/AMPK/mTOR/autophagy signaling and inhibiting osteosarcoma progression [55].

### 3.6. Study Limitations

This is a retrospective computational study using publicly available data. The identified E3 ligases should thus be interpreted as hypothesis-generating candidates associated with patient survival, not as experimentally validated drivers of sarcoma biology or proteosome addiction. As soft tissue sarcomas represent a broad and diverse group of cancer types, subtype heterogeneity is expected and could limit the breadth of applicability of associations discovered using the available TCGA data. Our violin plot data suggest that for the E3 ligases of interest in this study, most of the sarcomas evaluated show relatively even distribution of patients into high and low expression groups. The exceptions noted should be considered before applying conclusions for validation or patient prognosis.

Our initial survival analyses assessed all available cutoff values between the lower and upper quartiles of expression for each selected gene. This method can maximize effect size and reveal biologically meaningful inflection points that an arbitrary cutoff such as median would miss. However, because this cutoff is data-driven, it can inflate false-positive results and can be difficult to replicate in validation cohorts. To minimize acceptance of false positives, we limited substrate enrichment analyses to the E3 ligases that also had FDR values of 5% or less. We also performed survival analyses using median expression as the cutoff. This ensured that high and low expression groups were of equal size and protected against “*p*-value hacking” and overfitting. When median expression was used, associations with OS remained significant for four of the six E3 ligases initially associated with shortened OS and two of the six E3 ligases initially associated with extended OS with an FDR of ≤5%. Only one E3 ligase was associated with decreased RFS by both cutoff methods, and none were found to associate with extended RFS when median expression was used as cutoff.

## 4. Materials and Methods

### 4.1. Kaplan–Meier Survival Analysis

To identify E3 ubiquitin ligases that influence patient survival, we used Kaplan–Meier Plotter (https://kmplot.com/analysis/index.php?p=home, last accessed on 24 June 2026) [56,57] to produce survival curves for sarcoma patients with high and low expression of each E3 human ubiquitin ligase on the list curated by the epithelial systems biology laboratory (ESBL) in the National Heart Lung and Blood Institute (Supplemental Document S1) [21]. Kaplan–Meier Plotter curates patient data from GEO, EGA, and TCGA databases, including TCGA-housed data from a study of 259 soft tissue sarcoma patients [22]. Kaplan–Meier Plotter performed Cox proportional hazards regression and computed false-discovery rates. To avoid missing correlations due to the use of a specific cutoff such as median, all available cutoff values between the lower and upper quartiles of expression were assessed for each selected gene. False-discovery rates (FDR) were computed using the Benjamini–Hochberg method to correct for multiple hypothesis testing. For each E3 ligase, the cutoff value with the highest significance and lowest FDR was chosen. In cases in which multiple cutoff values yielded identical significance and FDR, the cutoff that yielded the highest hazard ratio (HR) was selected. Patient data included mRNA-sequencing based gene expression data, relapse-free survival, and OS. For sensitivity analysis, we performed independent Kaplan–Meier analyses of the impact of each E3 ubiquitin ligase found to significantly increase or decrease OS or RFS by >40 months using overall and relapse-free survival data for the 206 patients studied by Abehouse et al. [22]. This study excluded 22 cases with mRNA expression data that had been sent for genomic analysis but failed QC during annotation and 31 cases excluded in pathology review and included 80 leiomyosarcoma (53 soft tissue and 27 uterine), 50 dedifferentiated liposarcoma, 44 undifferentiated pleomorphic sarcoma, 17 myxofibrosarcoma, 10 synovial sarcoma, and 5 malignant peripheral nerve sheath tumor patients. Median transcripts per million (TPM) values for each assessed E3 ligase were set as cutoff to split tumor samples into “High” and “Low” expressing groups. The R package “survminer” [58] was used to generate Kaplan–Meier plots. *p*-values were generated using log-rank tests. E3 ligases with significant impacts on OS or RFS using median as cutoff are indicated with asterisks in column 1 of Table 1, Table 2, Table 3 and Table 4 (* *p* ≤ 0.05, ** *p* ≤ 0.01, *** *p* ≤ 0.001).

### 4.2. Violin Plot Analyses

To determine whether specific sarcoma types were segregating into high or low E3 ligase expression groups, we performed violin plot analyses using ggplot [59] in R on the 206 samples included by Abehouse et al. [22] to visualize expression of each E3 ligase in each patient with patients grouped in columns by sarcoma type (DDLPS = dedifferentiated liposarcoma; MFS = myxofibrosarcoma; MPNST = malignant peripheral neural sheath tumor; SS = synovial sarcoma; STLMS = soft tissue leiomyosarcoma; ULMS = uterine leiomyosarcoma; UPS = undifferentiated pleomorphic sarcoma).

### 4.3. Identification of Known and Predicted E3 Ligase Substrates

We employed UbiBrowser 2.0 [23] to identify known and putative substrates of each E3 ubiquitin ligase whose upregulation was found to correlate significantly with an OS increase or decrease of greater than 40 months. We then manually filtered out duplicates and substrates found in both datasets (substrates of ligases associated with extended OS and those associated with shortened OS).

### 4.4. Pathway Enrichment Analyses

Two sets of proteins were generated using UbiBrowser 2.0 that represent potential substrates uniquely associated with the six E3 ubiquitin ligases for which upregulated expression was associated with extended OS, and the six E3 ubiquitin ligases for which upregulated expression was associated with shortened OS. Each set of proteins was manually filtered to remove shared genes and then subjected to enrichment analysis using Reactome [60,61]. Reactome curates 15,886 reactions that function within 2803 human pathways. The pathways are hierarchically organized under 29 broad pathway categories: autophagy, cell cycle, cell–cell communication, cellular responses to stimuli, chromatin organization, circadian clock, developmental biology, digestion and absorption, disease, DNA repair, DNA replication, drug ADME, extracellular matrix organization, gene expression, hemostasis, immune system, metabolism, metabolism of proteins, metabolism of RNA, muscle contraction, neuronal system, organelle biosynthesis and maintenance, programmed cell death, protein localization, reproduction, sensory perception, signal transduction, transport of small molecules, and vesicle mediated transport.

### 4.5. Statistical Analyses

Kaplan–Meier Plotter uses a PostgreSQL server to integrate gene expression and clinical data. For any given gene of interest, the relevant set of patient records was split into high and low expression groups as described above. A Kaplan–Meier survival plot was then created, and a hazard ratio with 95% confidence intervals and logrank *p*-value were calculated. In enrichment analyses using Reactome, *p*-values associated with enriched functions were calculated using Fisher’s exact test.

## 5. Conclusions

Our analyses of E3 ligase impacts on survival among soft tissue sarcoma patients reveal small subsets of E3 ligases that shorten and extend OS and RFS. With a small number of caveats, these subsets may broadly serve as signatures that could aid in assessing patient prognosis across a broad range of soft tissue sarcoma types. Substrate identification and enrichment analyses provide insights into possible mechanisms for both shortening and extension of survival and may suggest new avenues of research for development of therapeutic options with narrower specificities and reduced risk of serious adverse events.

## Figures and Tables

**Table 1:** E3 ubiquitin ligases whose upregulation was associated with shortened overall survival by ≥40 months among soft tissue sarcoma patients.

E3 Ubiquitin Ligase	*p*-Value	FDR (%)	HR (95%CI)	N Low/N High	ΔOS (Months)
PCGF3 *	0.011	>50	1.66 (1.09–2.51)	181/78	44.66
CCNB1IP1	0.01	>50	1.64 (1.08–2.49)	189/70	40.43
MSL2 *	0.0087	>50	1.67 (1.1–2.53)	194/65	43.76
RNF2	0.0072	>50	1.68 (1.13–2.5)	162/97	40.3
MARCHF5 **	0.005	50	1.69 (1.13–2.52)	158/101	44.66
ZBTB12	0.002	20	1.94 (1.27–2.96)	194/65	43.97
MEX3D *	0.0016	20	1.86 (1.23–2.82)	116/143	40.93
UBE4B **	0.00013	2	2.20 (1.45–3.33)	194/65	40.27
TRIM71	0.00087	1	1.80 (1.18–2.74)	190/69	47.03
RNF8 ***	0.000045	1	2.46 (1.62–3.73)	195/64	44.93
MEX3A **	0.000023	1	2.26 (1.5–3.4)	133/126	45.46
MARCHF10	0.00001	1	1.96 (1.31–2.91)	160/99	48.63
TTC3 **	5.2 × 10^−8^	1	2.78 (1.87–4.14)	183/76	50.63

E3 ligases are listed by *p*-value in descending order. Asterisks indicate E3 ligases that yielded significant *p*-values using median expression level as threshold. * *p* ≤ 0.05, ** *p* ≤ 0.01, *** *p* ≤ 0.001. FDR = false-discovery rate; HR = hazard ratio; ΔOS = difference in overall survival.

**Table 2:** E3 ubiquitin ligases whose upregulation was associated with extended overall survival by ≥40 months among soft tissue sarcoma patients.

E3 Ubiquitin Ligase	*p*-Value	FDR (%)	HR (95%CI)	N High/N Low	ΔOS (Months)
RNF144B	0.03	>50	0.65 (0.43–1)	194/65	40.43
DTX3L	0.007	>50	0.59 (0.39–0.89)	190/69	42.43
RNF25	0.0066	50	0.61 (0.41–0.9)	169/90	40.3
SYVN1	0.0054	50	0.56 (0.37–0.86)	193/66	44.66
MARCHF1	0.0039	50	0.59 (0.39–0.87)	174/85	40.43
MARCHF8	0.0034	50	0.54 (0.35–0.82)	194/65	44.5
MARCHF2	0.003	50	0.55 (0.36–0.84)	194/65	40.43
ZNRF2	0.0028	20	0.55 (0.36–0.82)	179/80	40.06
BIRC3	0.0023	20	0.54 (0.36–0.82)	192/67	45.83
RNF135	0.0016	20	0.53 (0.36–0.8)	170/89	44.4
PCGF5	0.0013	20	0.53 (0.35–0.81)	185/74	44.66
TMEM129	0.0008	10	0.51 (0.34–0.76)	177/82	44.5
RMND5B **	0.00036	5	0.49 (0.32–0.75)	112/147	40.53
TRIM69	0.00011	1	0.47 (0.31–0.7)	188/71	44.4
TRIM22	0.000062	1	0.45 (0.3–0.68)	194/65	46.76
HERC6	0.000027	1	0.45 (0.3–0.67)	179/80	46.03
TRIM21 ***	0.000011	1	0.42 (0.28–0.64)	135/124	50.63
VPS18	9.4 × 10^−6^	1	0.43 (0.29–0.64)	167/92	50.86

E3 ligases are listed by *p*-value in descending order. Asterisks indicate E3 ligases that yielded significant *p*-values using median expression level as threshold. ** *p* ≤ 0.01, *** *p* ≤ 0.001. FDR = false-discovery rate; HR = hazard ratio; ΔOS = difference in overall survival.

**Table 3:** E3 ubiquitin ligases whose upregulation was associated with shortened recurrence-free survival among soft tissue sarcoma patients.

E3 Ubiquitin Ligase	*p*-Value	FDR (%)	HR (95%CI)	N Low/N High	ΔRFS (Months)
UBR5	0.041	>50	1.76 (1.01–3.07)	55/97	3.07
RNF138	0.034	>50	1.85 (1.04–3.29)	51/101	4.70
MEX3C	0.015	>50	2.05 (1.13–3.7)	46/106	7.67
RNF26 **	0.039	>50	1.68 (1.02–2.75)	104/48	56.37
MKRN2	0.047	>50	1.64 (1–2.69)	77/75	61.10
TRAF4 *	0.041	>50	1.65 (1.02–2.68)	87/65	68.66
TRIM36	0.045	50	1.63 (1.01–2.65)	88/64	61.10
BIRC2	0.012	50	1.88 (1.14–3.09)	105/47	69.70
RAD18	0.0077	20	1.95 (1.18–3.21)	77/75	6.77
SH3RF1	0.0035	20	2.04 (1.25–3.34)	89/63	7.80
RFWD3	0.0055	20	2.31 (1.26–4.23)	50/102	57.00
UHRF2	0.0066	10	1.96 (1.2–3.22)	81/71	5.8
TRAIP	0.0027	10	2.26 (1.31–3.89)	61/91	68.53
MNAT1	0.00046	2	2.40 (1.45–3.98)	113/39	75.03
UHRF1 ***	0.00027	1	2.48 (1.5–4.1)	113/39	72.30

E3 ligases are listed by *p*-value in descending order. Asterisks indicate E3 ligases that yielded significant *p*-values using median expression level as threshold. * *p* ≤ 0.05, ** *p* ≤ 0.01, *** *p* ≤ 0.001. FDR = false-discovery rate; HR = hazard ratio; ΔRFS = difference in recurrence-free survival.

**Table 4:** E3 ubiquitin ligases whose upregulation was associated with extended recurrence-free survival among soft tissue sarcoma patients.

E3 Ubiquitin Ligase	*p*-Value	FDR (%)	HR (95%CI)	N High/N Low	ΔRFS (Months)
TRIM8	0.037	>50	0.59 (0.36–0.97)	111/41	1.87
RBCK1	0.046	>50	0.61 (0.37–1)	107/45	48.84
RNF44	0.043	>50	0.58 (0.34–0.99)	114/38	56.37
UBE3B	0.0197	>50	0.56 (0.34–0.92)	103/49	57.20
RNF25	0.027	>50	0.56 (0.34–0.94)	112/40	57.77
RNF125	0.03	>50	0.58 (0.35–0.96)	71/81	58.83
VPS41	0.011	50	0.54 (0.33–0.87)	94/58	57.20
RMND5B *	0.01	50	0.53 (0.32–0.86)	104/48	58.64
MARCHF8	0.03	50	0.56 (0.33–0.96)	114/38	69.70
MGRN1	0.0088	20	0.52 (0.32–0.86)	103/49	57.20
RNF130	0.0041	10	0.49 (0.3–0.81)	83/69	70.03
TMEM129	0.00048	2	0.43 (0.26–0.7)	91/61	72.10

E3 ligases are listed by *p*-value in descending order. Asterisks indicate E3 ligases that yielded significant *p*-values using median expression level as threshold. * *p* ≤ 0.05. FDR = false-discovery rate; HR = hazard ratio; ΔRFS = difference in recurrence-free survival.

**Table 5:** Top significantly enriched functions among known and predicted substrates of E3 ubiquitin ligases whose upregulation decreases overall survival among soft tissue sarcoma patients.

Enrichment Term	Impact on Cancer	# of Proteins (%)	*p*-Value	FDR
CD22 mediated BCR regulation	Tumor suppressing	60 (83.33)	1.11 × 10^−16^	3.33 × 10^−15^
Packaging Of Telomere Ends	Tumor suppressing	25 (75.76)	1.11 × 10^−16^	3.33 × 10^−15^
DNA methylation	Cancer promoting	25 (71.43)	1.11 × 10^−16^	3.33 × 10^−15^
FCGR activation	Context dependent	70 (67.96)	1.11 × 10^−16^	3.33 × 10^−15^
Classical antibody-mediated complement activation	Context dependent	64 (65.98)	1.11 × 10^−16^	3.33 × 10^−15^
HDACs deacetylate histones	Cancer promoting	30 (47.62)	1.11 × 10^−16^	3.33 × 10^−15^
Regulation of endogenous retroelements by Piwi-interacting RNAs (piRNAs)	Context dependent	31 (45.59)	1.11 × 10^−16^	3.33 × 10^−15^
Role of LAT2/NTAL/LAB on calcium mobilization	Cancer promoting	65 (60.75)	1.11 × 10^−16^	3.33 × 10^−15^
FXIIa activates plasma kallikrein-kinin system	Cancer promoting	26 (61.90)	1.11 × 10^−16^	3.33 × 10^−15^
SIRT1 negatively regulates rRNA expression	Tumor suppressing	26 (59.09)	1.11 × 10^−16^	3.33 × 10^−15^
Condensation of Prophase Chromosomes	Tumor suppressing	29 (53.70)	1.11 × 10^−16^	3.33 × 10^−15^
RUNX1 regulates genes involved in megakaryocyte differentiation and platelet function	Context dependent	32 (42.11)	1.11 × 10^−16^	3.33 × 10^−15^
ERCC6 (CSB) and EHMT2 (G9a) positively regulate rRNA expression	Cancer promoting	27 (57.45)	1.11 × 10^−16^	3.33 × 10^−15^
Inhibition of DNA recombination at telomere	Tumor suppressing	26 (55.32)	1.11 × 10^−16^	3.33 × 10^−15^
Immunoregulatory interactions between a Lymphoid and a non-Lymphoid cell	Cancer promoting	120 (48.19)	1.11 × 10^−16^	3.33 × 10^−15^
Meiotic recombination	Cancer promoting	30 (50.85)	1.11 × 10^−16^	3.33 × 10^−15^
Cell surface interactions at the vascular wall	Cancer promoting	93 (36.19)	1.11 × 10^−16^	3.33 × 10^−15^
FCERI mediated NF-kB activation	Cancer promoting	64 (39.51)	1.11 × 10^−16^	3.33 × 10^−15^
Fc epsilon receptor (FCERI) signaling	Cancer promoting	70 (31.53)	1.11 × 10^−16^	3.33 × 10^−15^
Regulation of endogenous retroelements by the Human Silencing Hub (HUSH) complex	Tumor suppressing	25 (60.98)	1.11 × 10^−16^	3.33 × 10^−15^
FCERI mediated MAPK activation	Cancer promoting	67 (54.03)	1.11 × 10^−16^	3.33 × 10^−15^
FCGR3A-mediated IL10 synthesis	Cancer promoting	73 (51.77)	1.11 × 10^−16^	3.33 × 10^−15^
Fcg receptor (FCGR) dependent phagocytosis	Tumor suppressing	77 (39.49)	1.11 × 10^−16^	3.33 × 10^−15^
FCGR3A-mediated phagocytosis	Tumor suppressing	73 (46.50)	1.11 × 10^−16^	3.33 × 10^−15^
Adaptive Immune System	Tumor suppressing	215 (20.15)	1.11 × 10^−16^	3.33 × 10^−15^

Impact on cancer was assessed using AI mode in Google search, followed by individual assessment of provided references.

**Table 6:** Top significantly enriched functions among known and predicted substrates of E3 ubiquitin ligases whose upregulation increases overall survival among soft tissue sarcoma patients.

Enrichment Term	Impact on Cancer	# of Proteins (%)	*p*-Value	FDR
RNA Polymerase II Transcription	Cancer promoting	73 (4.35)	1.11 × 10^−16^	1.21 × 10^−13^
Generic Transcription Pathway	Cancer promoting	69 (4.38)	5.55 × 10^−16^	3.01 × 10^−13^
Gene expression (Transcription)	Cancer promoting	74 (3.85)	3.10 × 10^−14^	1.12 × 10^−11^
SUMO E3 ligases SUMOylate target proteins	Cancer promoting SUMOylate target proteins	20 (10.86)	1.58 × 10^−11^	4.28 × 10^−9^
SUMOylation	Cancer promoting	20 (10.36)	3.64 × 10^−11^	7.89 × 10^−9^
Transcriptional Regulation by TP53	Tumor suppressing	30 (6.16)	1.51 × 10^−10^	2.73 × 10^−8^
FOXO-mediated transcription	Tumor suppressing	15 (13.64)	2.82 × 10^−10^	4.37 × 10^−8^
Differentiation of naive CD4^+^ T cells to T helper 2 cells (Th2 cells)	Context dependent	13 (15.85)	7.49 × 10^−10^	1.01 × 10^−7^
Cytokine Signaling in Immune system	Context dependent	45 (4.07)	2.03 × 10^−9^	2.44 × 10^−7^
Activation of HOX genes during differentiation	Cancer promoting	14 (12.07)	5.17 × 10^−9^	4.89 × 10^−7^
Activation of anterior HOX genes in hindbrain development during early embryogenesis	Cancer promoting	14 (12.07)	5.17 × 10^−9^	4.89 × 10^−7^
PIP3 activates AKT signaling	Cancer promoting	22 (6.98)	5.43 × 10^−9^	4.89 × 10^−7^
Differentiation of T cells	Tumor suppressing	13 (13.00)	7.76 × 10^−9^	6.15 × 10^−7^
TP53 Regulates Transcription of Cell Cycle Genes	Tumor suppressing	11 (16.92)	7.99 × 10^−9^	6.15 × 10^−7^

Impact on cancer was assessed using AI mode in Google search, followed by individual assessment of provided references.

## Data Availability

Any data not provided in the manuscript and Supplementary Material will be made available upon request.

## Supplementary Materials

The following supporting information can be downloaded at: https://www.mdpi.com/article/10.3390/ijms27146350/s1.
