# Supplementary material for: Identification of E3 Ubiquitin Ligases Associated with Survival in Soft Tissue Sarcomas
Source: Int J Mol Sci. 2026 Jul 17;27(14):6350. doi: 10.3390/ijms27146350 (PMC13410252; doi:10.3390/ijms27146350)
Supplement: Supplementary file 1 [file ijms-27-06350-s001.zip › ijms-4364297 Supplementary Materials figures.pdf]

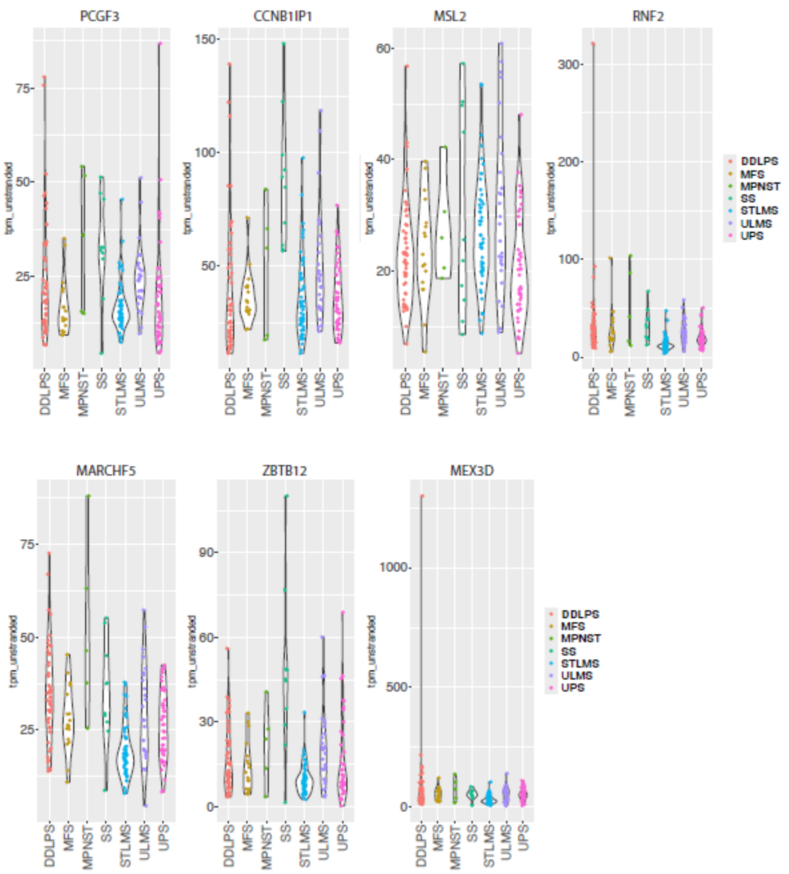

Figure S1. Violin plots of transcripts per million (TPM) values (Y-axis) for E3 ubiquitin ligases whose up regulation was associated with shortened overall survival among sarcoma patients by >40 months and FDR ≥ 5% according to sarcoma histologic type (Table 1). DDLPS = dedifferentiated liposarcoma; MFS = myxofibrosarcoma; MPNST = Malignant Peripheral Neural Sheath Tumor; SS = Synovial Sarcoma; STLMS = Soft tissue Leiomyosarcoma; ULMS = Uterine Leiomyosarcoma; UPS = Undifferentiated Pleomorphic Sarcoma

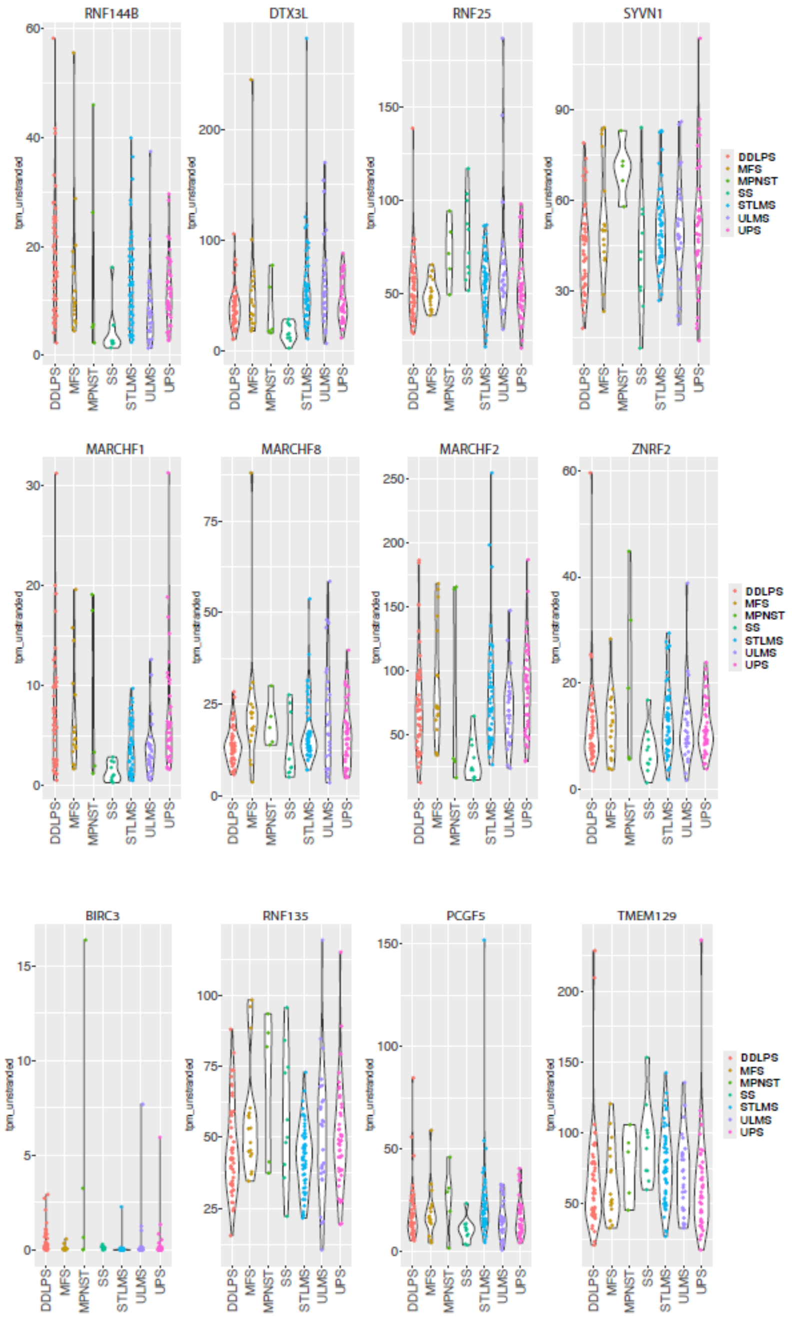

Figure S2. Violin plots of TPM values (Y-axis) for E3 ubiquitin ligases whose upregulation was associated with extended overall survival among sarcoma patients by >40 months and FDR ≥ 5% according to sarcoma histologic type (Table 2). DDLPS = dedifferentiated liposarcoma; MFS = myxofibrosarcoma; MPNST = malignant peripheral neural sheath tumor; SS = synovial sarcoma; STLMS = soft tissue leiomyosarcoma; ULMS = uterine leiomyosarcoma; UPS = undifferentiated pleomorphic sarcoma.

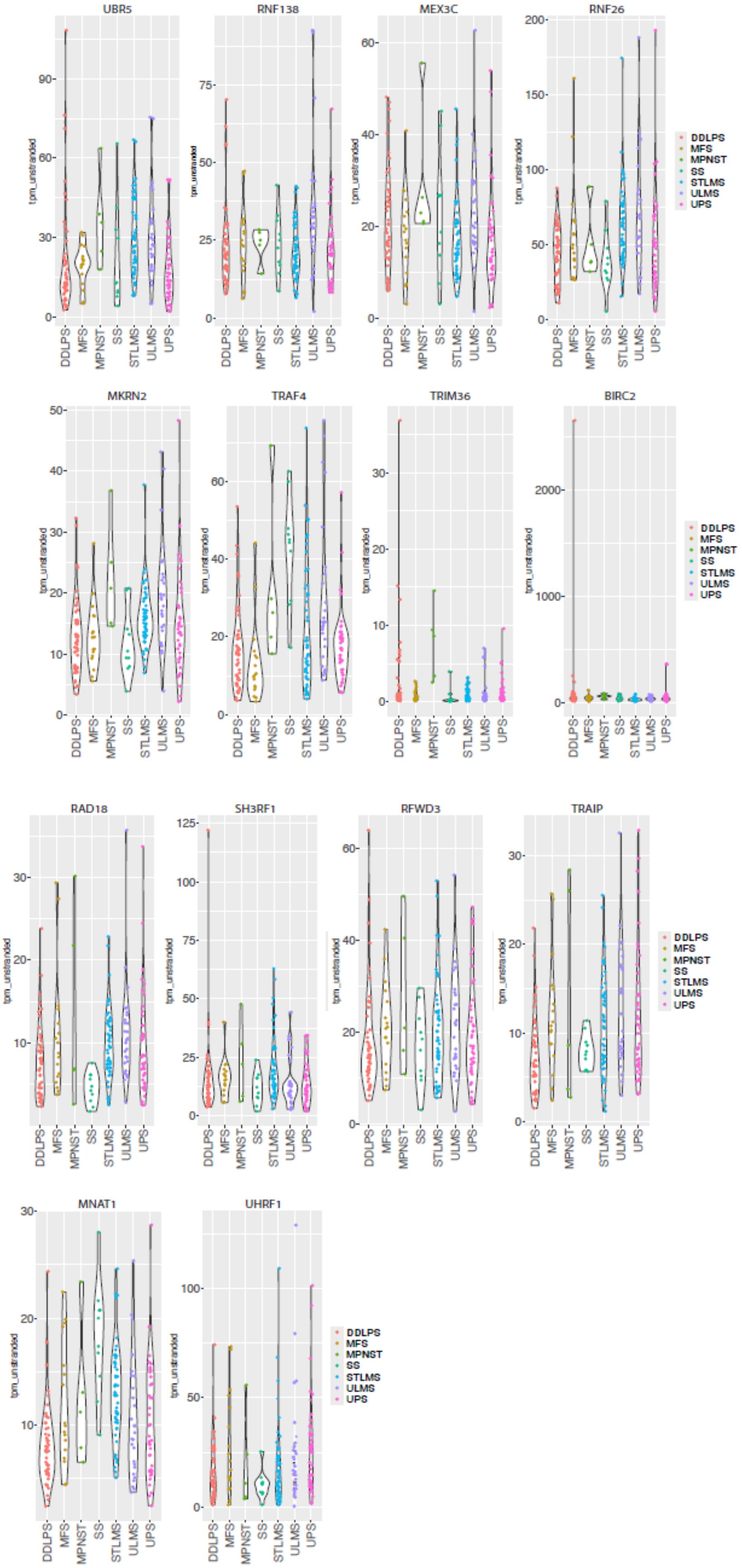
UBR5
RNF138
MEX3C
RNF26
MKRN2
TRAF4
TRIM36
BIRC2
RAD18
SH3RF1
RFWD3
TRAIP
MNAT1
UHRF1
tpm_unstranded
DDLPS
MFS
MPNST
SS
STLMS
ULMS
UPS

Figure S3. Violin plots of TPM values (Y-axis) for E3 ubiquitin ligases whose upregulation was associated with shortened recurrence free survival among sarcoma patients according to sarcoma histologic type (Table 3). DDLPS = dedifferentiated liposarcoma; MFS = myxofibrosarcoma; MPNST = malignant peripheral neural sheath tumor; SS = synovial sarcoma; STLMS = soft tissue leiomyosarcoma; ULMS = uterine leiomyosarcoma; UPS = undifferentiated pleomorphic sarcoma.

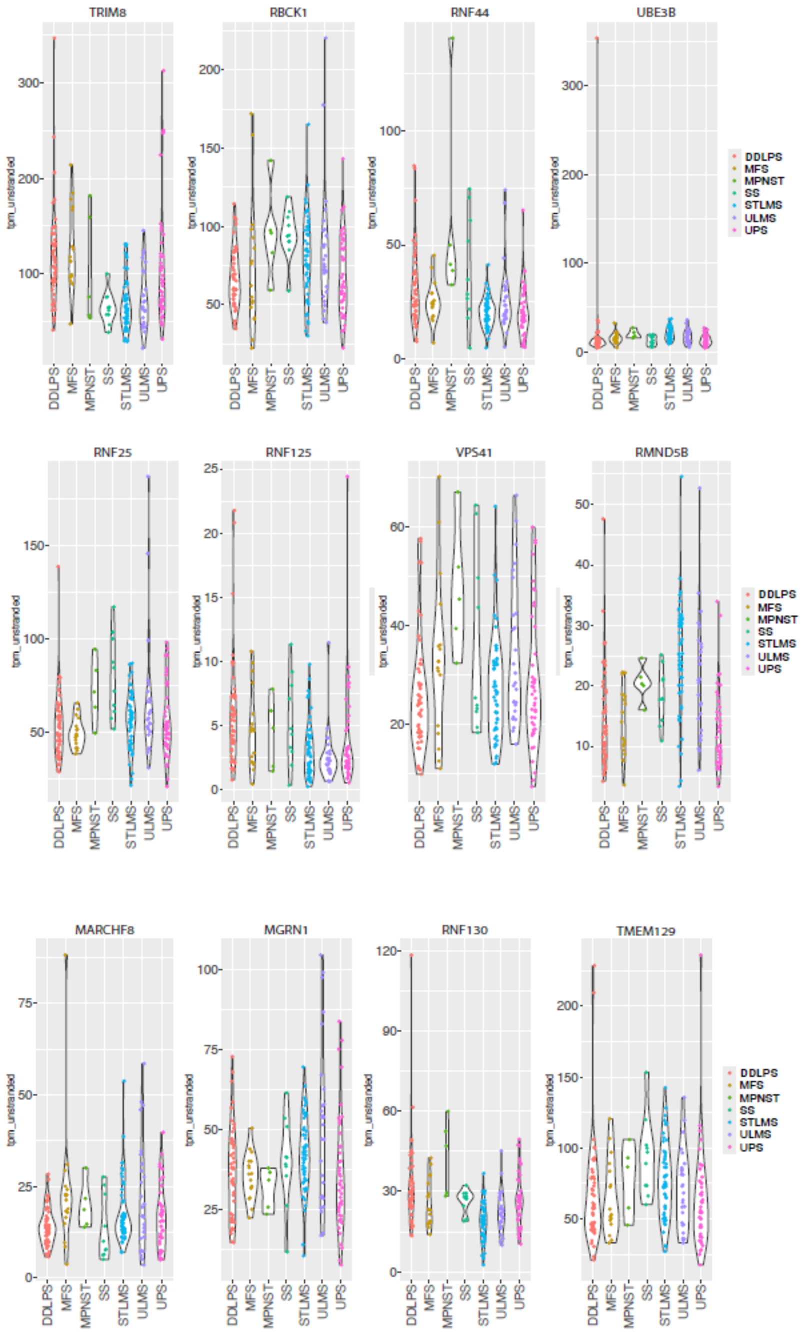

Figure S4. Violin plots of TPM values (Y-axis) for E3 ubiquitin ligases whose upregulation increases recurrence free survival among sarcoma patients according to sarcoma histologic type (Table 3). DDLPS = dedifferentiated liposarcoma; MFS = myxofibrosarcoma; MPNST = malignant peripheral neural sheath tumor; SS = synovial sarcoma; STLMS = soft tissue leiomyosarcoma; ULMS = uterine leiomyosarcoma; UPS = undifferentiated pleomorphic sarcoma.
